# Supplementary material for: Prediction of stunting and its socioeconomic determinants among adolescent girls in Ethiopia using machine learning algorithms
Source: PLoS One. 2025 Jan 24;20(1):e0316452. doi: 10.1371/journal.pone.0316452 (PMC11760002; doi:10.1371/journal.pone.0316452)
Supplement: Attachment — Submitted filename: Point by point response for reviewers.docx [file pone.0316452.s002.docx]

**April 1/2024**

**Point by point response for reviewer’s comments**

**Subject: - submission of revised manuscript and point by point response**

Manuscript Number: - PONE-D-23-41442

Title: “Application of Machine Learning-Based Algorithms for Predicting Stunting among Adolescent Girls in Ethiopia"

**To: - PLOS ONE** **Journal**

**Dear editor and reviewers,**

First of all, we would like to thank you for giving us the opportunity to made a revision and resubmit a revised manuscript. We appreciate the time and effort that you and the reviewers have dedicated to reviewing and providing constructive, building, and improvable comments that would improve the substance and content of the manuscript. We are very grateful for the insightful comments and valuable inputs to our manuscript. We have considered each comments and clarification questions of reviewer on the manuscript thoroughly. All comments, suggestions, and concerns of the reviewers are clearly stated and well-addressed in our point-by-point responses described in detail on the following pages. Furthermore, the details of changes were shown **using the red color** on the recently attached revised manuscript. The manuscript language was checked by language professionals and we have followed the journal guidelines upon preparation and submission of the manuscript. Thank you so much again!

Dear Dr. Zemariam,

Thank you for submitting your manuscript to PLOS ONE. After careful consideration, we feel that your manuscript will likely be suitable for publication if it is revised to address the points below. Therefore, my decision is "Major Revision".

Kind regards,

Oluwafemi Samson Balogun, Ph.D.

Academic Editor, PLOS ONE

Authors’ response: Dear respected academic editor; we thank you for your time, kind words, and special consideration to provide us valuable comments to enhance the quality of our manuscript. We have incorporated all comments, questions and suggestions raised by the reviewers. We kindly request you to see the point by point response and the revised manuscript.

Journal Requirements:

When submitting your revision, we need you to address these additional requirements.

1. Please ensure that your manuscript meets PLOS ONE's style requirements, including those for file naming. The PLOS ONE style templates can be found at

https://journals.plos.org/plosone/s/file?id=wjVg/PLOSOne_formatting_sample_main_body.pdf and

<https://journals.plos.org/plosone/s/file?id=ba62/PLOSOne_formatting_sample_title_authors_affiliations.pdf>

Authors’ response: Dear we thank you and greatly appreciate your valuable suggestion. Rest assured, we have carefully reviewed our manuscript to ensure it complies with the guidelines set by your premier journal.

2. We noticed you have some minor occurrence of overlapping text with the following previous publication(s), which needs to be addressed:

https://bmcmedinformdecismak.biomedcentral.com/articles/10.1186/s12911-023-02102-w

https://www.mdpi.com/2227-9067/10/10/1638

<https://archpublichealth.biomedcentral.com/articles/10.1186/s13690-015-0093-9>

In your revision ensure you cite all your sources (including your own works), and quote or rephrase any duplicated text outside the methods section. Further consideration is dependent on these concerns being addressed.

Authors’ response: Dear we sincerely thank you for your diligence in identifying the minor occurrence of overlapping text with the previous publication(s). We greatly appreciate your efforts in bringing this matter to our attention. Ensuring the integrity and originality of our work remains our top priority, and we apologize for any inconvenience caused. We have fully accepted your valuable comment, revised the manuscript accordingly, promptly taken action to rectify it, and provided appropriate citations. We kindly invite you to review the revisions made in the updated manuscript.

3. Your ethics statement should only appear in the Methods section of your manuscript. If your ethics statement is written in any section besides the Methods, please move it to the Methods section and delete it from any other section. Please ensure that your ethics statement is included in your manuscript, as the ethics statement entered into the online submission form will not be published alongside your manuscript.

Authors’ response: Dear we thank you for your crucial recommendation. We have accepted and revised it accordingly. Kindly see the corrections made in the revised manuscript.

4. Please include a separate caption for each figure in your manuscript.

5. Please include captions for your Supporting Information files at the end of your manuscript, and update any in-text citations to match accordingly. Please see our Supporting Information guidelines for more information: http://journals.plos.org/plosone/s/supporting-information.

Authors’ response: We sincerely appreciate your valuable suggestions. We have thoroughly reviewed and accepted them, and have been incorporated into the revised manuscript. We kindly request you to review the updated version of the manuscript to see the changes made in response to your vital recommendation.

Reviewers' comments:

Reviewer's Responses to Questions

Comments to the Author

1. Is the manuscript technically sounds, and do the data support the conclusions?

Reviewer #1: No

Reviewer #2: Yes

2. Has the statistical analysis been performed appropriately and rigorously?

Reviewer #1: No

Reviewer #2: Yes

3. Have the authors made all data underlying the findings in their manuscript fully available?

Reviewer #1: Yes

Reviewer #2: Yes

4. Is the manuscript presented in an intelligible fashion and written in standard English?

Reviewer #1: No

Reviewer #2: Yes

5. Review Comments to the Author

Please use the space provided to explain your answers to the questions above.

**Reviewer #1: Introduction could potentially be enhanced:**

1. Expanding on how machine learning has been utilized in public health, especially in developing countries, could provide a more comprehensive backdrop. This includes examples of successful ML applications in similar contexts.

Authors’ response: Dear respected reviewer thank you for your imperative suggestion. We agree that providing a more comprehensive backdrop would enhance the quality of our manuscript. In response to your comment, we have accepted and revised the manuscript accordingly. We kindly request you to review the revised manuscript to see the expanded content.

2. A detailed discussion about the challenges in predicting stunting, especially in low-resource settings, and how machine learning can address these challenges would be informative. This includes limitations of traditional statistical methods and the advantages offered by ML techniques.

Authors’ response: Dear reviewer, we thank you for your valuable suggestion. We agree that exploring the limitations of traditional statistical methods and highlighting the advantages offered by machine learning techniques would provide informative insights. We have accepted and expanded the introduction in the revised manuscript to delve into the challenges of stunting prediction, the shortcomings of traditional statistical approaches, and the potential benefits of utilizing machine learning methods in this context. We encourage you to review the updated manuscript to see the revisions made.

3. For readers who may not be familiar with machine learning, a clearer explanation of key ML concepts and algorithms could be beneficial. This includes a basic overview of the algorithms used in the study and why they are suitable for this type of analysis.

Authors’ response: Dear esteemed reviewer, thank you for your vital suggestion. In response to your comment, we have included a more concise explanation of the concepts of machine learning and explained why or based on what criteria we have applied some selected algorithms in the introduction section of the revised manuscript. Moreover, we kindly request you to see the details of the advantage and limitation of each algorithm under the methods section.

4. How stunting is influenced by socio-economic and environmental factors, and how ML can help untangle these complex relationships, would provide a more holistic understanding.

Authors’ response: Dear reviewer, we thank you for your concrete comment. We have thoroughly addressed your issue in the revised manuscript. Hence, we have expanded to highlight your important issue.

**Firstly**, stunting is influenced by socio-economic and environmental factors:

#. Malnutrition: Inadequate access to quality food, especially during critical growth periods, contributes to stunting. Chronic malnutrition is often associated with impoverished socio-economic conditions.

#. Poverty: Families in poverty struggle to provide nutritious food, clean water, and healthcare, leading to higher rates of stunting.

#. Maternal health and nutrition: Malnourished mothers are more likely to give birth to underweight babies at higher risk of stunting. Maternal factors like low height and inadequate prenatal care also contribute.

#. Sanitation and hygiene: Poor sanitation and lack of clean water increase the risk of infections and diseases, impairing nutrient absorption and increasing nutrient requirements.

#. Education and awareness: Lack of knowledge about nutrition, childcare, and hygiene practices contributes to stunting.

#. Healthcare access: Limited access to healthcare facilities, including prenatal care and treatment for illnesses, can contribute to stunting.

#. Environmental factors: Pollution, toxins, and poor air quality can negatively impact child growth and development.

Therefore, addressing stunting requires comprehensive measures, including improving socio-economic conditions, promoting education and awareness, enhancing healthcare services, and improving sanitation and hygiene practices.

**Secondly**, machine learning can contribute to unraveling these intricate relationships between socio-economic and environmental factors in stunting by:

A. Feature Selection: Identifies key factors and discards irrelevant variables.

B. Nonlinear Relationships: Captures complex interactions and dependencies.

C. Prediction and Risk Assessment: Predicts stunting likelihood and assesses combined effects of factors.

D. Data Integration: Combines diverse data to uncover hidden patterns.

E. Identifying Complex Interactions: Reveals how factors interact, e.g., income, water access, and education.

F. Targeted Interventions: Identifies at-risk subpopulations for tailored interventions. Machine learning enhances understanding for evidence-based interventions and policies against stunting.

5. Providing a review of previous studies that have used ML for similar purposes, highlighting what has been achieved and where gaps remain

Authors’ response: Dear esteemed reviewer, we thank you for your important comment. We have incorporated a comprehensive review of relevant literature, outlining the key findings, advancements achieved through the application of machine learning and existing gaps and associated limitations. Kindly refer to the revised manuscript.

6. Rationale for the study, particularly the need for ML approaches in the context of Ethiopia.

Authors’ response: Dear esteemed reviewer, we express our gratitude for your concrete comment. We have taken your comments into careful consideration and made the necessary revisions to the manuscript. We appreciate the value you have added through your insightful remarks and kindly invite you to review the revisions incorporated in the updated manuscript. Thank you once again for your valuable input.

Other drawbacks

7. The absence of some potentially relevant clinical, dietary, and socio-economic variables in the DHS data may affect the accuracy and comprehensiveness of the results.

Authors’ response: Dear reviewer, we appreciate your concern and thank you for your nice issue. The authors acknowledged that due to secondary source of date (DHS) some important variables might be missed which have an impact on the model predictive accuracy and comprehensiveness of our results. Therefore, we recommended that future researcher should strive to include a broader range of relevant variables to improve the model prediction using data set other than DHS.

8. The study focuses specifically on adolescent girls in Ethiopia, which may limit the generalizability of the findings to other populations or demographic groups.

Authors’ response: Dear honored reviewer, we sincerely appreciate your concern. While our study provides specific insights into adolescent girls' in Ethiopia, caution should be exercised when applying the results to other contexts. Therefore, we recommend that future researchers should include a broader range of populations other than adolescent girls to improve generalizability and deepen our understanding of the topic.

9. The Boruta algorithm, employed for feature selection in the study, is a robust method that ensures comprehensive inclusion of relevant features. However, it comes with drawbacks such as high computational intensity due to its iterative nature and reliance on random forest classifications, which can be resource-heavy. There's a risk of over fitting, as Boruta tends to retain more features, potentially including noisy or less relevant ones. Its dependence on the performance of the random forest algorithm means its effectiveness is closely tied to how well this model fits the data. Additionally, the process of feature selection can sometimes be arbitrary and lacks clear interpretability, as the reasons for the inclusion or exclusion of certain features aren't always transparent. Furthermore, the efficiency of Boruta can be challenged in handling very high-dimensional data due to the exponential increase in features when creating shadow attributes.

Authors’ response: Dear esteemed reviewer, we sincerely appreciate your critical comment. We completely agree with your point. While Boruta is indeed a powerful tool for feature selection, there can be potential challenges associated with its usage. In our study, we have explored various feature selection methods such as Lasso method, PCA; wrapper methods include forward selection, backward elimination, and recursive feature elimination, correlation-based feature selection, and chi-square test and compared their performance using evaluation metrics to select the best feature selection. Through this analysis, we have found that Boruta is the most effective feature selection method. Furthermore, we acknowledge the challenges posed by Boruta as a limitation of the study. Thank you once again for your valuable comment and kindly see it on the revised manuscript. Here below are some challenges and possible solution taken in our manuscript.

A. High computational cost: The algorithm requires multiple iterations and comparisons with shadow features, which can increase the processing time. Therefore, to mitigate this issue the authors considered reducing the number of iterations or subsampling the dataset. Besides, we utilized parallel processing or distributed computing techniques to speed up the computation.

B. Sensitivity to hyper parameters: Boruta has a few hyper parameters that need to be set, such as the number of random shadow features to create and the threshold for feature importance. To address this challenge, authors had performed hyper parameter tuning using techniques like cross-validation or grid search, or Bayesian optimization, or random search.

C. Handling correlated features: Boruta assumes that features are independent of each other, but in reality, there may be correlations among the features. To address this problem, we had conducted principal component analysis (PCA) and algorithms that explicitly handle feature dependencies, such as recursive feature elimination with cross-validation to identify and reduce the impact of correlated features.

D. Interpretability of results: Boruta provides a ranking of feature importance, but it does not explicitly provide information on the relationship between features and the target variable. To solve this issue, after applying Boruta authors had conducted additional analysis like data visualization, statistical tests, or domain knowledge to interpret the selected features and gain insights into the relationship between the selected features and the target variable.

E. Limited applicability to certain types of data: Boruta is primarily designed for supervised learning tasks, where the target variable is known. It may not be suitable for unsupervised or semi-supervised learning problems. Therefore, we believed that our task was used a supervised machine learning, therefore, Boruta has to be the most appropriate choice than clustering-based methods or manifold learning techniques. Thank you once again for your thoughtful comment.

F. Over fitting: We have implemented several techniques to address this limitation. These techniques include using L1 or L2 regularization, employing cross-validation, maintaining an independent test set, analyzing feature importance stability across multiple runs or subsets, and applying conservative feature selection criteria. Therefore, by incorporating these precautions, we have tried to reduce the risk or limitation associated with Boruta algorithm to enhance the generalization and performance of the selected features and model prediction.

10. Machine Learning Model Interpretability can be seen as "black boxes". Include interpretable AI

Authors’ response: Dear reviewer, we deeply appreciate your valuable comment. Machine learning models have faced criticism due to their lack of interpretability, being perceived as opaque predictors. To address this, interpretable AI has emerged, aiming to develop models that not only achieve accurate predictions but also offer explanations. By incorporating techniques like rule-based models and feature importance analysis, we can gain insights into the decision-making process of the model and identify potential biases.

Furthermore, we acknowledge the challenge of addressing the "black box" nature of the Random Forest Classifier. To overcome this, we have done the following strategies in our analysis:

A. Analyzing feature importance scores to focus on the top-ranking features and gain insights into their impact on predictions.

B. Visualizing the decision paths and rules in the ensemble's decision trees to understand the model's decision-making process.

C. Prioritizing interpretability, we have simplified the Random Forest model by reducing the number of trees or using only the most important features.

D. Applying model explanation techniques like SHAP analysis to provide local or global explanations for individual predictions and gain a detailed understanding of the model's behavior.

E. Considering alternative models such as decision trees or linear models that inherently offer more transparency. Dear esteemed reviewer, by employing these strategies we have gained a better understanding of how the model works and the relative importance of different features and we are confident that these have enhanced our understanding and the relative importance of different features on model prediction by overcoming the limitation of the Boruta and RF algorithm. We have taken into account this issue within the limitation of our study and provide further insight for future researchers.

11. ML models parameter optimization is missing

Authors’ response: Dear esteemed reviewer, thank you for raising this very important issue. We apologizes you for missing this very important section and now we have included in the revised manuscript. This additional information enhances transparency and reproducibility, allowing readers to understand and replicate our approach. We appreciate your valuable feedback, which has strengthened the rigor and transparency of our study. Kindly see the revised manuscript.

12. Include a flowchart to explain the study clearly

Authors’ response: Dear reviewer, we thank you for your suggestion to enhance the clarity of our study. In response to your suggestion, we have incorporated a comprehensive flowchart in the revised manuscript. Kindly see it in the updated version of our manuscript.

13. Despite using the Synthetic Minority Oversampling Technique (SMOTE) to balance the data, there's a risk that this technique might introduce artificial bias. Oversampling can sometimes lead to over fitting, as the model might be too tailored to the synthetic examples created by SMOTE. Explain this.

Authors’ response: Dear reviewer, we thank you for your insightful questions. We have thoroughly explored various methods to address class imbalance, including ensemble approaches that combine multiple sampling techniques such as over sampling, under sampling, ADASYN, and SMOTE. By comparing these methods, we determined that SMOTE demonstrated the best performance in our analysis. Kindly see the revised manuscript.

Moreover, to overcome the risks of bias and over fitting in SMOTE, we have done the following:

A. Evaluate the SMOTE impact in our dataset.

B. Experiment with different nearest neighbor’s values to reduce bias.

C. Validate using cross-validation for a more robust assessment.

D. Apply regularization techniques like L1 or L2 regularization, dropout, or early stopping.

E. Consider collecting more data for the minority class to reduce the need for oversampling. While SMOTE is commonly employed, it is important to acknowledge its potential limitations. To address these concerns, we applied careful model evaluation techniques and acknowledged the limitations associated with SMOTE techniques. We emphasize the need for further research on larger and more diverse datasets to validate our findings. Additionally, exploring many more alternative methods or ensemble approaches could help mitigate the risk of over fitting and improve the generalization performance of the models.

14. Missing Data percentage

Authors’ response: Dear esteemed reviewer, thank you for your insightful comment, which we greatly appreciate. We have carefully examined various approaches for managing missing data in machine learning, including deletion methods (list wise and feature-wise), imputation techniques (mean, median, mode, hot deck, regression, and multiple imputation), model-based imputation (using KNN, or random forests), and leveraging domain-specific knowledge. Taking into account the nature of the missingness, the amount of missing data, the underlying data assumptions, and the specific machine learning algorithm employed, we have opted to handle missing values in our dataset using the K-nearest neighbor (KNN) imputation technique. K-nearest neighbor (KNN) imputation offers several benefits, including the ability to retain all available data by not removing instances or features with missing values, resilience to outliers due to its reliance on nearby neighbors rather than the overall data distribution, the absence of assumptions about the missingness mechanism, applicability to both numerical and categorical features, adaptability to new data points or dataset changes without the need for retraining, and the capacity to encompass a broader spectrum of potential values while minimizing the introduction of bias into the imputed values. In our data the missing value was less than 20% of the total data set. Many scholars recommend KNN imputation as it is a more effective handling technique when the percentage of missing values is relatively low to moderate, typically below 30% of the total dataset. Moreover, we have taken precautions to ensure that this method does not introduce bias or distort the underlying patterns in the data. Additionally, we have assessed the impact of different missing data techniques on the performance of our model through rigorous cross-validation and other evaluation methods.

15. The absence of p-values in the summary statistics table of the study could be considered a drawback, particularly in the context of medical and epidemiological research.

Authors’ response: Dear reviewer, we thank you so much for your concrete comment. We appreciate your observation regarding the absence of p-values in the summary statistics table of our study, especially considering the context of medical and epidemiological research. In the context of machine learning, the reporting of p-values is not a common practice, and it is not typically employed because we prioritized predictive performance rather than hypothesis testing and p-value calculation. Besides, the aim of ML is to learn patterns and relationships in the data to make accurate predictions rather than providing statistical inference. But, if it is must to be done and reported, we can incorporate it in the revised manuscript alongside machine learning techniques, unless we have removed it from the limitation section of our revised manuscript.

16. Results interpretation please indicate the clearly the results is based on testing dataset.

Authors’ response: Dear esteemed reviewer, we appreciate your comment. We apologize for any confusion caused in our presentation. In order to address this concern, we have revised and ensured that the results section to explicitly state that the reported findings are based on the evaluation of the model using the testing dataset. Thank you for bringing this to our attention.

17. The study uses association rule mining to identify patterns between features and stunting. However, these rules should be interpreted with caution as they indicate correlation, not causation, and the findings might be influenced by confounding factors.

Authors’ response: Dear reviewer, we appreciate your cautionary note regarding the interpretation of association rules in our study. We have checked the multicollinearity issue using the VIF method. While efforts were made to control for confounders, the possibility of residual or unmeasured confounding remains. We acknowledged these limitations and recommended to conduct further research using rigorous methodologies to investigate causal relationships and better understand the underlying mechanisms. By doing so, we can gain a more comprehensive understanding of the factors influencing stunting and make informed decisions to address this public health issue.

18. The discussion section of the manuscript could benefit from a more explicit with a broader and more detailed comparison with existing literature. Addressing the study's methodological limitations more thoroughly would provide a more balanced view. There's also a need for a more comprehensive discussion on potential biases inherent in the dataset and how these might influence the findings, a more detailed exploration of specific policy recommendations and practical applications would add value. Suggestions for future research, especially addressing the current study's limitations, could provide clearer direction for subsequent studies. Finally, a more in-depth discussion of the socioeconomic, cultural, and environmental factors affecting stunting in the Ethiopian context would offer more understanding of the issue.

Authors’ response: Dear respected reviewer, we appreciate your valuable feedback on the discussion section of our manuscript. We agree that providing a broader and more detailed comparison with existing literature would enhance the context and relevance of our findings. Additionally, addressing the study's methodological limitations more thoroughly would contribute to a more balanced interpretation.

We acknowledge the need for a comprehensive discussion on potential biases inherent in the dataset and their potential influence on the findings. Exploring specific policy recommendations and practical applications derived from our study would indeed add value and help bridge the research-to-practice gap. Furthermore, we understand the importance of suggesting future research directions that address the limitations of our current study. By providing clearer guidance for subsequent studies, we can contribute to the advancement of knowledge in this area.

We have taken these suggestions into account and revise the discussion section accordingly to enhance its comprehensiveness, address limitations, provide clearer policy implications, and suggest future research directions. Thank you for your insightful recommendations, kind words, and concrete comments which will improve the overall quality of the manuscript.

**Reviewer #2:**

The manuscript contains an important insight for handling and exploring the non-linear relationship of variables, it is an excellent supplement to results generated using the classical linear statistical models. In general, the manuscript is interesting and relevant to the field.

The following issues needs your reflection and where necessary revisions.

1. Do you have a rationale to split the dataset to 80% and 20% for training and testing the model respectively, why not other proportions?

Authors’ response: Dear respected reviewer, we thank you for your kind word and concrete comment. The 80-20 split is commonly used and has become a widely used default split in machine learning, but it's not a strict rule. The choice of split ratio can depend on factors like dataset size, problem complexity, and desired model performance. Alternative proportions like 70-30 or 90-10 can be considered. For instance, if the dataset is very large, a 90-10 split might still provide sufficient training data while increasing the testing set's size. Conversely, a 70-30 split might be chosen if the dataset is limited, and more data is needed for training. Moreover, techniques like cross-validation and stratified sampling can also be utilized in our analysis to enhance model evaluation. Ultimately, the split ratio should be determined based on the specific requirements and constraints of the problem, as well as empirical experimentation to find the optimal balance between training and testing data. Additionally, in our study we have used the 80-20 splitting techniques for several reasons:

A. Sufficient Training Data: To capture underlying patterns and relationships, it is important to have a large training set. By allocating 80% of the data for training, the model has enough data to learn from and generalize effectively.

B. Effective Model Evaluation: The testing set is used independently to assess the model's performance. Allocating 20% of the data for testing ensures that the model is evaluated on unseen data, preventing over fitting. It provides a substantial amount of data for assessing the model's generalization ability.

C. Statistical Significance: The 80-20 split strikes a balance between having sufficient training data and a significant evaluation set. This split has been proven to work well in various machine learning tasks and datasets, based on empirical evidence.

D. Empirical Success: Over time, the 80-20 split has gained wide acceptance and is commonly utilized in different machine learning domains. It has demonstrated practical effectiveness and is often the default choice in well-established machine learning frameworks and libraries.

2. What strategy has employed to mitigate over fitting in your study

Authors’ response: Dear esteemed reviewer, we deeply appreciate your crucial comment. In order to address the issue of over fitting in our machine learning study, we have implemented a variety of strategies:

A. Augmenting the Training Data: We have augmented the training data to increase its size, which helps the model capture more underlying patterns and reduces the risk of over fitting.

B. Simplifying the Model: To minimize over fitting, we have simplified the model architecture by reducing the number of layers, neurons, or parameters. Additionally, we have employed regularization techniques like L1 or L2 regularization to prevent the model from fitting the training data too closely.

C. Leveraging Cross-Validation: Instead of relying solely on a single train-test split, we have utilized cross-validation techniques such as k-fold cross-validation. This approach provides a more robust estimation of the model's performance by evaluating it across multiple train-test splits.

D. Employing Early Stopping: By monitoring the model's performance on a validation set during training, we have implemented early stopping. This involves halting the training process when the model's performance on the validation set ceases to improve, thereby avoiding over-optimization on the training data.

E. Utilizing Regularization Techniques: We have incorporated regularization techniques, such as L1 or L2 regularization, which introduce penalty terms into the loss function. This discourages the model from fitting the training data too closely, thus controlling its complexity and mitigating over fitting.

F. Harnessing Ensemble Methods: To combat over fitting, we have leveraged ensemble methods like bagging or boosting. By combining multiple models, we can reduce over fitting and generate predictions that are more generalizable.

G. Conducting Feature Selection/Engineering: We have carefully selected relevant features and performed feature engineering to reduce noise and enhance the model's generalization capability.

H. Employing Train-Validation-Test Split: To ensure proper evaluation, we divided the dataset into three subsets: training, validation, and testing. The training set was used to train the model, the validation set was employed for hyper parameter fine-tuning and performance monitoring, and the testing set served to assess the final model performance.

Besides, we have experimented with fine-tune techniques based on the specific problem and dataset to achieve optimal performance and mitigate over fitting effectively.

3. Have you used the same software for data preprocessing and model selection analysis, if not kindly mention which other tools were employed?

Authors’ response: Dear reviewer, we appreciate your feedback on this matter. In response to your query, we confirm that we utilized the same software (python) for both data preprocessing and model selection analysis in our study. The software employed was a widely utilized and comprehensive machine learning framework that encompassed functionalities for both of these tasks. This approach allowed us to streamline our workflow and maintain consistency throughout the entire process.

For data preprocessing, we leveraged popular Python libraries such as Pandas, NumPy, and Scikit-learn. These libraries provided a wide range of capabilities for tasks such as data manipulation, handling missing values, feature scaling, encoding, and selection.

In terms of model selection analysis, Python offered a plethora of libraries that facilitated the evaluation and selection of models. Notable libraries include Matplotlib and Seaborn, Keras, PyTorch, and XGBoost, among others. These libraries provided various algorithms, techniques for hyper parameter tuning, and evaluation metrics to assess and compare different models effectively. By utilizing this combination of Python libraries, we were able to accomplish both data preprocessing and model selection tasks within a unified and efficient framework. Moreover, we utilized R studio to perform Boruta algorithm. Kindly see the revised manuscript.

4. You have mentioned a number of performance evaluation metrics, why don’t you use one of the metrics that best fit to your case.

Authors’ response: Dear respected reviewer, thank you for raising this issue. In our study, we carefully considered the nature of our problem, the specific task, and the characteristics of our data to select the most suitable evaluation metric. For instance, if the problem at hand involved imbalanced classes, we might have placed a stronger emphasis on metrics like precision, recall, or F1-score, which provide insights into the model's ability to handle class imbalance. On the other hand, if the problem required a balanced consideration of both positive and negative instances, accuracy and AUC could be a more appropriate metric.

Although various evaluation metrics were presented in our study, we specifically employed AUC and accuracy as the primary metrics to assess the model's performance in our study. AUC and accuracy are widely recognized as valuable metrics for evaluating the effectiveness of models, especially in binary classification tasks. These metrics were chosen based on their established reputation and their ability to provide meaningful insights into model performance. The followings are some of the reasons for using these evaluation techniques among others.

A. Aggregates Performance across All Thresholds: AUC and accuracy assess the model's discrimination power across all possible thresholds, ensuring a comprehensive evaluation.

B. Robust to Class Imbalance: AUC and accuracy are reliable metrics for imbalanced datasets, accounting for both true positive and false positive rates.

C. Insensitive to Decision Threshold: AUC and accuracy remain unaffected by the choice of decision threshold, making them suitable when threshold variations occur or are unknown.

D. Interpretability and Comparability: AUC and accuracy provide interpretable scalar values, facilitating easy comparison of models or experiments.

Dear esteemed reviewer, we kindly ask you to see the revisions made in the revised manuscript.

5. Feature selection methods is not well explained, specifically it is important to depict how the employed feature selection method (Boruta algorithms) is suitable over other methods for your study.

Authors’ response: Thank you dear reviewer for bringing up this important issue. We apologize for the lack of clarity in explaining why we chose the Boruta algorithm for feature selection. We have addressed the comment in our revised manuscript and kindly request you to see the revision in the updated manuscript. Here are the key reasons why Boruta was considered suitable compared to the other many methods:

A. Ability to Handle Complex Relationships: Boruta is adept at capturing both linear and non-linear dependencies between features and the target variable, making it well-suited for datasets with intricate relationships.

B. Robustness to Noisy and Redundant Features: Boruta has a mechanism to identify and remove irrelevant features, distinguishing them from truly important ones. This enhances model efficiency and generalization capabilities.

C. No Assumptions about Feature Distribution: Boruta can be applied to datasets with non-normal or skewed feature distributions since it does not make any assumptions about the distribution of features.

D. Incorporation of Randomization: Boruta uses randomization to create shadow features for evaluating the importance of original features. This helps mitigate biases and provides a more robust assessment of feature importance.

E. Model Agnostic: Boruta is not tied to a specific machine learning model and can be used with various classifiers. It can be applied in different domains and problem contexts without relying on assumptions about the underlying model.

**Note**: Besides, we have employed different feature selection techniques to overcome the limitation or challenges associated with the Boruta algorithm and we carefully compared each technique and we selected the Boruta as a best feature selection method. Considering the complexity of the relationships in our dataset, the need to handle noise, and redundancy while identifying relevant features, these characteristics made Boruta a suitable choice for our study. We appreciate your feedback and hope this explanation clarifies why we selected Boruta as the feature selection method. Dear respected reviewer, we thank you and appreciate your attention and the valuable input you have provided.

6. The first paragraph of the discussion part is well explained on the introduction section, no need to repeat it, hence remove it and focus on interpreting your findings and portray its policy and further research implications.

Authors’ response: Dear reviewer, thank you for your valuable feedback. We have accepted and removed the repetition of the first paragraph from the discussion section and we focused on interpreting our findings and discussing their policy implications as well as suggesting avenues for further research. Kindly see the revision in the revised manuscript.

7. Random forest algorithm has the best predictive model in your case; your discussion is limited to studies that come across with similar result to yours. But there are studies which identify best predictive model other than random forest algorithm, you have to include them and narrate in relation to your findings.

Authors’ response: Dear esteemed reviewer, thank you for your valuable suggestion and we sincerely appreciate your positive feedback. We acknowledge the existence of studies that have identified predictive models other than the random forest algorithm as the best in their respective cases. In response, we have revised our discussion to incorporate these studies and provide a comparative analysis in relation to our own findings. We kindly request you to review the revisions made in the revised manuscript.

8. Paragraphs narrating the predictors of stunting and the use of association rule mining on the discussion part is direct replication of the result and devoid of interpretation and comparison with other studies’ findings.

Authors’ response: Dear respected reviewer, we thank you for your very insightful view. We apologize for the direct replication of the results. We have revised these paragraphs to include more interpretation and comparison with findings from other relevant studies. We kindly request you to see the revised manuscript.

#. PLOS authors have the option to publish the peer review history of their article (what does this mean?). If published, this will include your full peer review and any attached files.

If you choose “no”, your identity will remain anonymous but your review may still be made public.

Do you want your identity to be public for this peer review? For information about this choice, including consent withdrawal, please see our Privacy Policy.

Reviewer #1: Yes: Sorayya Malek

Reviewer #2: No
