# Supplementary material for: Prediction of stunting and its socioeconomic determinants among adolescent girls in Ethiopia using machine learning algorithms
Source: PLoS One. 2025 Jan 24;20(1):e0316452. doi: 10.1371/journal.pone.0316452 (PMC11760002; doi:10.1371/journal.pone.0316452)
Supplement: Attachment — Submitted filename: Review report.docx [file pone.0316452.s003.docx]

**Review report**

Review report of research manuscript entitled **“Application of Machine Learning-Based Algorithms for Predicting Stunting among Adolescent Girls in Ethiopia”**

I would like to say thank you for your invitation to review the above-mentioned manuscript!

General Comments

The paper is technically much sounded and the questions are informative. The statistical analysis of the data is also highly sounded, and the findings are appropriately discussed in the context of previous literature.  It meets the requirements of the research community well in the data processing and management steps.

1. Reporting prevalence in the summary section for this particular study is senseless. You are running out of the scope of your title. Try to remove it please.
2. Starting from the title you should identify and incline towards either the model or the application mainly. Based on that the title, background, objectives, and all others will be shaped in that way! As to me, the ultimate objective looks emphasizing the application. My suggested topic/title: “Prediction of stunting and its socioeconomic determinants among adolescent girls in Ethiopia using machine learning algorithms”
3. Why are you interested to study only adolescent girls? Give a strong justification in your introduction. Do you think that stunting is the only concern of adolescent girls?
4. What was the mechanism that can be used to manage the lack of transparency and interpretability of the data?
5. What were the possible bias and limitations for your study? In addition, how could you handle it? Try to incorporate these issues in your study clearly.
6. Why did you exclude other parts of undernutrition? Why do you insisted to stunting only? Please give a strong justification.
7. Some of very relevant factors of stunting cannot be available in the secondary data including DHS data. How did you handle such issues? Try to incorporate in your limitation.
8. Eight machine-learning algorithms were included for model building and comparison for this particular study. What additional models did you plan in your future studies? What challenges did you face to be restricted to these eight models only for this study? What are the major effects for your study regarding variations of machine learning algorithms?
9. EDHS 2016 is old aged data. Why did you prefer it? There are recent EDHS data in other countries. Have you consider this issue?
10. To keep the logical coherence and flow of ideas, I recommend you to mention the consequences and effects of stunting following to the prevalence of stunting.
11. Why did you re categorize wealth index?
12. I appreciate your mean SHAP value report and Waterfall plot analysis. However, I have not seen any interpretation for such wonderful parts of your effort. I strongly recommend you to interpret it in detail using the log odd value for each specific findings to make it clear for readers.
13. Make sure that all of your pertinent findings have been discussed very well including its implications.
14. Artificial intelligence is the current active area of research particularly for health data. You have particularly employed machine learning for this specific study. Why did you prefer machine-learning parts of artificial intelligence on top of other artificial intelligence options like generative artificial intelligence and deep learning?
15. Try to revise editorial and grammatical issues throughout your entire document.

Generally, this research addressed untouched and active research area using multiple machine learning algorithms and large data set. After correcting the given comments, this study is qualified for publication, potentially contribute significant role for the entire scientific community.
