# Supplementary material for: Prediction of stunting and its socioeconomic determinants among adolescent girls in Ethiopia using machine learning algorithms
Source: PLoS One. 2025 Jan 24;20(1):e0316452. doi: 10.1371/journal.pone.0316452 (PMC11760002; doi:10.1371/journal.pone.0316452)
Supplement: Attachment — Submitted filename: Response to Reviewer.docx [file pone.0316452.s004.docx]

**September 27/2024**

**Point by point response for reviewer’s comments**

**Subject: - submission of revised manuscript and point by point response**

Manuscript Number: - PONE-D-23-41442R1

Title: “Application of Machine Learning-Based Algorithms for Predicting Stunting among Adolescent Girls in Ethiopia"

**To: - PLOS ONE** **Journal**

**Dear editor and reviewers,**

First of all, we would like to thank you for giving us the opportunity to made a revision and resubmit a revised manuscript. We appreciate the time and effort that you and the reviewers have dedicated to reviewing and providing constructive, building, and improvable comments that would improve the substance and content of the manuscript. We are very grateful for the insightful comments and valuable inputs to our manuscript. We have considered each comments and clarification questions of reviewer on the manuscript thoroughly. All comments, suggestions, and concerns of the reviewers are clearly stated and well-addressed in our point-by-point responses described in detail on the following pages. Furthermore, the details of changes were shown **using the red colour** on the recently attached track change revised manuscript. The manuscript language was checked by language professionals and we have followed the journal guidelines upon preparation and submission of the revised manuscript. Thank you so much again!

Dear Dr. Zemariam,

Thank you for submitting your manuscript to PLOS ONE. After careful consideration, we feel that your manuscript will likely be suitable for publication if it is revised to address the points below. Therefore, my decision is "Minor Revision".

**Journal Requirements:**

Please review your reference list to ensure that it is complete and correct. If you have cited papers that have been retracted, please include the rationale for doing so in the manuscript text, or remove these references and replace them with relevant current references. Any changes to the reference list should be mentioned in the rebuttal letter that accompanies your revised manuscript. If you need to cite a retracted article, indicate the article’s retracted status in the References list and also include a citation and full reference for the retraction notice.

**Authors’ response**: Thank you for your constructive feedback on our reference list. We have thoroughly reviewed all cited sources. Thankfully, we did not find any retracted references. We appreciate your attention to this issue and your insights, which have contributed to the improvement of our manuscript.

**General Comments**

The paper is technically much sounded and the questions are informative. The statistical analysis of the data is also highly sounded, and the findings are appropriately discussed in the context of previous literature.  It meets the requirements of the research community well in the data processing and management steps.

1. Reporting prevalence in the summary section for this particular study is senseless. You are running out of the scope of your title. Try to remove it please.

**Authors’ response**: Dear esteemed reviewer, we thank you for your suggestion. We have accepted the comment and removed it accordingly.

2. Starting from the title you should identify and incline towards either the model or the application mainly. Based on that the title, background, objectives, and all others will be shaped in that way! As to me, the ultimate objective looks emphasizing the application. My suggested topic/title: “Prediction of stunting and its socioeconomic determinants among adolescent girls in Ethiopia using machine learning algorithms”

**Authors’ response:** Dear Reviewer, thank you for your helpful comments on our manuscript's focus. We have revised the title to: “Prediction of Stunting and Its Socioeconomic Determinants among Adolescent Girls in Ethiopia Using Machine Learning Algorithms.” Additionally, we have adjusted the background and objectives to emphasize the application and practical implications of our findings. Your feedback has greatly improved our work. Thank you again for your valuable input.

3. Why are you interested to study only adolescent girls? Give a strong justification in your introduction. Do you think that stunting is the only concern of adolescent girls?

**Authors’ response**: Dear Reviewer, thank you for your valuable feedback regarding our focus on adolescent girls. When we see the global as well as the national burden of stunting its more common in both the under-five particularly young children whose age is less than two years and adolescent girls due to several reasons:

A. Rapid Growth Phases: Young Children: During early childhood, rapid growth requires adequate nutrition; deficiencies can lead to stunting. Adolescent Girls: This group experiences significant growth spurts, making them vulnerable to nutritional deficiencies.

B. Nutritional Needs: Both young children and adolescent girls have increased nutritional needs for growth and development. Insufficient intake of essential nutrients can hinder growth.

C. Socioeconomic Factors: Many adolescent girls and young children come from disadvantaged backgrounds, facing food insecurity and limited access to healthcare, which increases the risk of stunting.

D. Cultural Practices: In some cultures, girls may receive less nutritional support than boys, leading to higher stunting rates among girls.

E. Health Issues: Young children are more susceptible to infections and illnesses, which can impair nutrient absorption and contribute to stunting.

F. Menstrual Health: Adolescent girls may experience additional challenges related to menstruation, such as increased iron needs, which, if unmet, can affect overall health and growth.

While numerous studies have focused on young children, our research did not uncover any predictions of stunting specifically among adolescent girls. We believe that adolescent girls are especially vulnerable to the impacts of stunting, which can lead to long-term health and developmental issues. Implementing targeted interventions and evidence-based policies can help address the intergenerational burden of malnutrition. Although stunting is not the only concern for this group, we emphasize its importance as a critical issue that can significantly affect their future health and opportunities plus the coming generation health and well beings. Thank you for encouraging us to improve the clarity and depth of our manuscript.

4. What was the mechanism that can be used to manage the lack of transparency and interpretability of the data?

**Authors’ response**: Dear Reviewer, thank you for your important question regarding the management of transparency and interpretability of the data in our study.

To address these concerns, we have implemented the following mechanisms:

A. Model Selection: We have chosen interpretable machine learning models, such as decision trees or logistic regression, which provide clearer insights into how predictions are made.
B. Feature Importance Analysis: We have conducted a feature importance analysis to highlight which variables most significantly influence stunting predictions. This helps clarify the relationship between predictors and outcomes.
C. Visualization Tools: We have utilized visualization techniques, such as SHAP (SHapley Additive exPlanations) values, to illustrate the contributions of individual features to the model's predictions, enhancing interpretability.
D. Documentation and Reporting: Comprehensive documentation of our methodology and findings has been included in the manuscript to ensure transparency in our data handling and analysis processes.

These strategies help to enhance the interpretability of our findings and ensure that our conclusions are accessible and actionable. Again thank you for your concern and valuable feedback.

5. What were the possible bias and limitations for your study? In addition, how could you handle it? Try to incorporate these issues in your study clearly.

**Authors’ response**: Dear reviewer, we thank you for your insightful question regarding the possible biases and limitations of our study.

**Top Possible Biases and Limitations**:

A. Data Quality and Accuracy: Secondary datasets may contain inaccuracies due to self-reported data, leading to potential measurement bias that could impact model performance.
B. Temporal Relevance: The 2016 dataset may not adequately represent current conditions or trends, limiting the applicability of our findings to today’s context.
C. Missing Data: The presence of missing values in key variables can reduce the robustness of the analysis and lead to biased predictions.
D. Feature Selection Bias: The variables included in the dataset may not capture all relevant factors influencing stunting, leading to incomplete model interpretations.
E. Overfitting: Machine learning models can become overly complex, fitting noise in the data rather than the underlying patterns, especially with a limited dataset.

**Handling These Issues**:

A. Data Cleaning: We have performed a comprehensive assessment of the dataset to identify and address inaccuracies, ensuring data quality before analysis.
B. Contextual Limitations: We have clearly discussed the limitations associated with using the 2016 data in our manuscript, including implications for generalizability.
C. Imputation Techniques: We have applied appropriate imputation methods to handle missing data, minimizing its impact on our analysis.
D. Robust Feature Selection: We have carefully selected and justified the features included in our models, potentially incorporating additional relevant variables where possible.
E. Model Validation: We have used techniques such as cross-validation to assess model performance and prevent overfitting, ensuring that our findings are reliable.

We have clearly articulated these biases and limitations, along with our strategies for addressing them, in the revised manuscript. Your feedback has been instrumental in guiding these improvements. Thank you for your constructive input.

6. Why did you exclude other parts of undernutrition? Why do you insisted to stunting only? Please give a strong justification.

**Authors’ response**: Dear Reviewer, thank you for your thoughtful question regarding our focus on stunting while excluding other aspects of undernutrition.

**Rationale for Focusing on Stunting**:

**High burden**: several studies shown adolescent girls are more vulnerable to stunting compared to underweight and wasting for several key reasons:

A. Growth Patterns: Stunting reflects chronic nutritional deficiency over time, particularly during critical growth periods. Adolescence is marked by significant physical and hormonal changes, making adequate nutrition essential for achieving optimal height.
B. Nutritional Requirements: During adolescence, girls experience increased nutritional needs due to growth spurts and the onset of menstruation. If these needs are not met consistently, it can lead to stunting rather than short-term weight loss (wasting) or being underweight.
C. Long-Term Impact: Stunting has long-term implications for health and development, affecting cognitive function and future reproductive health. In contrast, underweight and wasting are often more acute conditions that may not have the same lasting consequences if addressed promptly.
D. Cultural and Socioeconomic Factors: In many societies, girls may have less access to nutritious food compared to boys, especially during adolescence. This disparity can lead to chronic undernutrition, resulting in stunting rather than immediate weight loss.
E. Health Complications: Chronic health issues, such as anemia or infections, can contribute to stunting more significantly than they do to underweight or wasting, as these conditions hinder growth over time.

Therefore, by concentrating on stunting, we aim to develop targeted interventions that address this specific form of undernutrition, which is crucial for improving health outcomes in adolescent girls which aligns with current global health priorities, including the Sustainable Development Goals (SDGs), which emphasize reducing stunting as a key indicator of nutritional status. While we recognize the importance of other forms of undernutrition, our decision to focus on stunting allows us to provide a detailed and actionable analysis that can inform targeted policies and interventions. Kindly see the revisions made in the revised manuscript and again thank you for prompting us to clarify this aspect in our manuscript.

7. Some of very relevant factors of stunting cannot be available in the secondary data including DHS data. How did you handle such issues? Try to incorporate in your limitation.

**Authors’ response**: Dear respected reviewer, we thank you for your insightful question regarding the limitations of using the DHS dataset, which may not include all relevant factors related to stunting.

**Handling Missing Factors**:

A. Acknowledgment of Limitations: We have explicitly acknowledged in the revised manuscript that the DHS dataset may lack certain important factors (e.g., dietary diversity, clinical or household food security) that could influence stunting.
B. Selection of Available Variables: We have focused on the variables that are present in the dataset and are known to be significant predictors of stunting. This will allow us to create a robust analysis while recognizing the gaps.
C. Suggestions for Future Research: We recommend that future studies incorporate additional data sources or primary data collection to capture these missing factors and provide a more comprehensive understanding of stunting.
D. Contextual Interpretation: We have discussed how the absence of these factors may limit the generalizability of our findings and emphasize the need for cautious interpretation of the results.

By incorporating these points in our limitations section, we aim to provide a clearer understanding of the dataset's constraints and the implications for our analysis. Your feedback has been invaluable in refining our approach. Thank you once again for your constructive input.

8. Eight machine-learning algorithms were included for model building and comparison for this particular study. What additional models did you plan in your future studies? What challenges did you face to be restricted to these eight models only for this study? What are the major effects for your study regarding variations of machine learning algorithms?

**Authors’ response**: Dear Reviewer, we thank you for your insightful questions regarding the machine-learning algorithms used in our study.

**Future Models**: In future studies, we plan to explore additional models such as:

A. Deep Learning Models: Neural networks, particularly for complex interactions in larger datasets.
B. Ensemble Methods: Techniques like Random Forests or Gradient Boosting Machines to improve predictive accuracy.

**Challenges Faced**: The decision to limit our analysis to eight specific models was influenced by several factors:

A. Computational Resources: The available computational power constrained the number of models we could effectively train and evaluate within the study timeframe.
B. Data Limitations: The size and quality of the dataset limited our ability to apply more complex models without risking overfitting.
C. Time Constraints: The need to thoroughly evaluate and compare models within the study's timeline restricted our scope.

**Major Effects on Our Study**: The choice of machine-learning algorithms can significantly affect the study's outcomes:

A. Predictive Performance: Different algorithms may yield varying levels of accuracy, impacting the reliability of our predictions.
B. Interpretability: Some models provide better interpretability, which is crucial for informing public health interventions and policy.
C. Generalizability: The selected algorithms influence how well our findings can be applied to different populations or settings.

We have incorporated these points into the revised manuscript to provide a comprehensive understanding of our modelling choices and their implications. We thank you again from the bottom of our heart for your critical points and crucial feedbacks.

9. EDHS 2016 is old aged data. Why did you prefer it? There are recent EDHS data in other countries. Have you considered this issue?

**Authors’ response**: Dear Reviewer, we thank you for your valuable question regarding our choice to use the 2016 EDHS data.

Rationale for Using 2016 EDHS Data:

A. Comprehensive Dataset: The 2016 EDHS dataset provides a rich and comprehensive set of variables specifically related to stunting and socioeconomic factors, which are crucial for our analysis.
B. Relevance to Study Objectives: At the time of our study, the 2016 data was the most recent dataset available in Ethiopia that included detailed information on adolescent girls, allowing us to address our research objectives effectively.
C. Benchmarking: Using this dataset allows us to establish a benchmark for understanding stunting trends over time, especially considering that previous datasets (e.g., 2011) are less comprehensive.

**Consideration of More Recent Data**:

We acknowledge that there are more recent EDHS datasets available in other countries. However, based on the previous classical studies and different organization reports the burden of stunting among adolescent girls are high in low income countries such as Ethiopia, that’s why our focus is Ethiopia and the specific context of adolescent girls led us to prioritize the 2016 dataset. Generally, we have accepted your vital comment and incorporate it as the limitation of the study. Thank you for prompting us to clarify this rationale in our manuscript.

10. To keep the logical coherence and flow of ideas, I recommend you to mention the consequences and effects of stunting following to the prevalence of stunting.

**Authors’ response**: Dear esteemed Reviewer, thank you for your constructive suggestion regarding the logical coherence of our manuscript. In response, we have revised the relevant sections to explicitly mention the consequences and effects of stunting immediately after its prevalence, enhancing the flow of ideas and clarifying its significance as a public health issue. We have outlined how stunting can lead to chronic health issues, such as increased susceptibility to infections and long-term developmental delays. Additionally, we have emphasized its impact on cognitive function and educational outcomes, which affect future opportunities for affected individuals. We have also discussed the broader socioeconomic implications, including reduced productivity and increased healthcare costs for communities and countries. By integrating these points, we aim to strengthen the manuscript's argument and provide a comprehensive view of the implications of stunting. Kindly see the revisions made in the updated manuscript of introduction section.

11. Why did you re categorize wealth index?

**Authors’ response**: Dear Reviewer, thank you for your question regarding the re-categorization of the wealth index in our study. We decided to re-categorize the wealth index to better align it with the specific socioeconomic context of our target population and improved statistical analysis or better model fit that can lead to more appropriate data distributions, improving the fit of statistical models and enhancing predictive accuracy. Besides, the original categories may not have adequately captured the nuances of wealth distribution relevant to stunting in adolescent girls.

12. I appreciate your mean SHAP value report and Waterfall plot analysis. However, I have not seen any interpretation for such wonderful parts of your effort. I strongly recommend you to interpret it in detail using the log odd value for each specific finding to make it clear for readers.

**Authors’ response**: Dear esteemed reviewer, we thank you for your thoughtful feedback and appreciate your recognition of these components of our work.

In response to your suggestion, we have included a detailed interpretation of the SHAP values, specifically focusing on the log odds for each significant finding. This helps us to clarify how each feature contributes to the model's predictions and enhance the overall understanding for readers and ensured this interpretation is comprehensive and accessible. Kindly see the revised manuscript on result section of model interpretability.

13. Make sure that all of your pertinent findings have been discussed very well including its implications.

**Authors’ response**: Dear respected reviewer, we thank you for your feedback on the discussion of our findings. We have ensured that all significant results and their implications are clearly articulated in the revised manuscript. We appreciate your guidance and we committed to improving the clarity and depth of the discussion.

14. Artificial intelligence is the current active area of research particularly for health data. You have particularly employed machine learning for this specific study. Why did you prefer machine-learning parts of artificial intelligence on top of other artificial intelligence options like generative artificial intelligence and deep learning?

**Authors’ response**: Dear esteemed reviewer, we thank you for your insightful question regarding my choice of machine learning for this study. I opted for machine learning techniques because they are particularly effective for analysing structured health data, allowing for interpretable results and robust performance with small datasets. While generative AI and deep learning have their merits, they often require more complex architectures and larger datasets, which may not align with the specific goals of this research..

**Generally, Reasons for Preferring Machine Learning over other AI parts are**:

Interpretability: Machine learning models, like decision trees, are more interpretable, which is crucial in fields like healthcare for trust and decision-making.

Data Requirements: Machine learning performs well with smaller datasets, making it practical when data is limited, unlike deep learning which requires large amounts.

Computational Efficiency: Machine learning models are less resource-intensive and quicker to train, making them accessible for many researchers.

Simplicity and Speed: They allow for faster prototyping and iteration compared to more complex deep learning models.

Task Suitability: For structured data tasks, traditional machine learning often yields effective results without the complexity of deep learning.

Robustness: Machine learning can be less prone to over fitting, especially with regularization techniques.

Ease of Implementation: Many practitioners are more familiar with machine learning, facilitating collaboration and knowledge sharing.

While generative AI and deep learning excel in unstructured data scenarios, machine learning offers a more straightforward, interpretable, and efficient approach, particularly where understanding and resource constraints are critical. However, we have recommended for future researcher to use a large complex dataset and utilize the other part of artificial intelligence like deep learning and generative AI.

15. Try to revise editorial and grammatical issues throughout your entire document.

Generally, this research addressed untouched and active research area using multiple machine learning algorithms and large data set. After correcting the given comments, this study is qualified for publication, potentially contribute significant role for the entire scientific community.

**Authors’ response**: Dear esteemed reviewer, we thank you for your positive feedback and appreciate your recognition of the study's contribution to an active and underexplored area. After addressing the provided comments, we believe this study will make a meaningful contribution to the scientific community. Besides, we thank you for your suggestion regarding the editorial and grammatical issues. We have carefully revised the entire document to address these concerns and ensure clarity and professionalism in the writing.
